# Children, CT Scan and Radiation

**Published:** 2010

**Authors:** Morteza Bajoghli, Farshad Bajoghli, Nazila Tayari, Reza Rouzbahani

**Affiliations:** 1 Department of Radiology, Al-Zahra Hospital, Isfahan University of Medical Sciences, Isfahan, Iran; 2 Department of Dental Prosthetics, School of Dentistry, Isfahan University of Medical Sciences, Isfahan, Iran; 3 Specialist in Community Medicine, Isfahan University of Medical Science, Isfahan, Iran

**Keywords:** Computer tomography, Magnetic Resonance Imaging, Millisievert, Radiation, Prevention

## Abstract

Children are more sensitive to radiation than adults. Computerized tomography (CT) consists of 25 % of all medical imaging. It was estimated that more than 2% of all carcinomas in the USA are due to CT scans. There is an ongoing focus on the reduction of CT scan radiation dose. Awareness about risk-benefits of CT has increased. Reduction of radiological exam is an important issue because the accumulation effects of radiation can be hazardous. In addition, proper protocol should be followed for diagnostic procedures of ionization radiation and computerized tomography. Effective radiation dose should range from 0.8 to 10.5 millisievert. The same protocol should be followed in different hospitals as well. Basic principles of radiation protection should be monitored. As much as possible, both technician and radiologist must be present during computerized tomography for children, and MRI and ultrasound should be replaced if possible.

## INTRODUCTION

Computed tomography (CT) scan in children may be used with caution and some considerations. However, its use has been increased seven times compared to 1980’s.^1^ Past studies showed that 50% of all CTs have high radiation dose and CTs consist of 25% of all medical imaging.^2^–^6^ It is estimated that more than 62 million CT scans per year are currently obtained in the United States, including at least 4 million for children.^7^ Children are considerably more sensitive to radiation than adults. As confirmed in epidemiologic studies of exposed populations, they have a longer life expectancy than adults resulting in a larger window of chance for expressing radiation damage. Finally, they obtain a higher dose than necessary when adult CT settings are used for kids.

In June 2009, World Health Organization (WHO) in an international conference on children’s health and environment in South Korea suggested the principles of “safe use of radiation in pediatrics”. At the same time, journal of radiology in April 2009 published an article about radiation dose, dose risk and dose reduction. There is an ongoing focus on reduction of radiation dose. In November 2007, “The New England Journal of Medicine” published an article by Brenner and Hall which stated that up to 2% of all carcinomas in the USA could be secondary to CT radiations.^7^ Advances in science and technology showed that the use of CT has its own indications.^8^ We should have enough knowledge about CT, so it will not be used improperly.^9^ Tube current modulation is an essential procedure for dose reduction especially for children.^10^ Increased number of detectors, change of detector technology, absorption source, isocenter distance and dose index changes for tube current, bring us to create a way to use appropriate reduction in dose with best quality of imaging in children.^11^ Singh and colleagues in their article, “Reduction of radiation dose in CT for children”, have suggested six different zones with different colors according to children’s weight and frequency of previous CTs. Six colorful zones consist of the followings:

1. Routine examination (pink zone) such as CT scan for appendicitis.
2. Follow up CT scans (green zone).
3. More follow up exams (red zone).
4. Exams that must be done in hospital setting (yellow zone) like kidney stone.
5. To detect congenital abnormalities (blue zone).
6. CT angiography (gray zone).

This protocol reduced radiation dose, however the important point is to keep the proper quality of CT, to avoid artifacts and to keep good image quality so that small structures could be visible. Also, low dose radiation prevents high CT noise. Therefore, special technique should be used and both technician and radiologist should be present during CT exams.^12^ In Pediatric Radiology in 2008, a model was proposed named “Image Gently Campaign” which means working together to change practice for better care and to perform CT exam in children’s hospital.^13^ In AJR in 2008, it was suggested to do MRI exams and ultrasounds instead of CT scans as much as possible. Also, we have to give special lectures for physicians who prescribe CT scan for children and to record CT orders in special computerized folders for future references.^14^ Variation in radiation dose in CT scan is surprising. According to Dr. G. Rubin from Stanford University, effective radiation dose should range from 0.8 to 10.5 mSv. Sometimes, effective dose may be over 10 mSv. He suspected that this was due to scanner, which is an older technology and no longer being used.^15^ In Radiological society of North America in 2009, Dr. Griffey from Washington University suggested that in emergency rooms with several CT exams, accumulation of radiation dose may have accumulating side effects leading to cancer.^16^ In pediatric nursery, with portable exams, scatter and transmission dose from repeated exposure must be measured.^17^ Rarely, a second malignant neoplasm in pediatric patients can develop in children with previous radiation therapy, chemotherapy or in those with genetic pre-disposition.^18^ In April 2009, Journal of Radiology described accumulative risk associated with recurrent CT exams. This news was published based on 245 new papers and was estimated that 149 million people were made aware of it.^19^ Since neonates and children are three to four times sensitive to ionization, radiation and CT, they should be aware of risk versus benefits of these exams.^20^ In September 2009, Radiological Society of North America (RSNA) described that doctors have to be familiar with strategies of reducing radiations to patients. Also, we have to explain the accumulation effects of radiation to the referring doctor and have to perform a cost-effective and safe procedure for the patients.^21^ American Association of Physicist in Medicine in Feb 2010 under the heading “tip of the month” described that typical abdominal CT scan has an effective dose of approximately equal to 2-3 years of natural background radiation in USA. This information emphasizes again the caution use of CT scan.^22^

## DISCUSSION

The risk of radiologic exam and CT seems to be lower than the natural risk although accumulation effect of radiation can be hazardous. Reduction of radiological exam is an important point. In addition, the proper protocol should be followed for diagnostic procedure by means of ionization radiation and CT. The same protocol should be followed in different hospitals as well. Basic principles of radiation protection should be observed. The number of procedures must be justified for lower radiation dose.

## CONCLUSION

As much as possible, both technician and radiologist must be present during CT for children, and MRI and ultrasound should be preferred over CT scan, specially for children and in out-patient clinics.
